# The effect of disease and respiration on airway shape in patients with moderate persistent asthma

**DOI:** 10.1371/journal.pone.0182052

**Published:** 2017-07-31

**Authors:** Spyridon Montesantos, Ira Katz, Jose Venegas, Marine Pichelin, Georges Caillibotte

**Affiliations:** 1 Medical R&D, Air Liquide Santé International, Paris Saclay, France; 2 Department of Mechanical Engineering, Lafayette College, Easton, PA, United States of America; 3 Massachusetts General Hospital and Harvard Medical School, Boston, MA, United States of America; Telethon Institute for Child Health Research, AUSTRALIA

## Abstract

Computational models of gas transport and aerosol deposition frequently utilize idealized models of bronchial tree structure, where airways are considered a network of bifurcating cylinders. However, changes in the shape of the lung during respiration affect the geometry of the airways, especially in disease conditions. In this study, the internal airway geometry was examined, concentrating on comparisons between mean lung volume (MLV) and total lung capacity (TLC). A set of High Resolution CT images were acquired during breath hold on a group of moderate persistent asthmatics at MLV and TLC after challenge with a broncho-constrictor (methacholine) and the airway trees were segmented and measured. The airway hydraulic diameter (Dh) was calculated through the use of average lumen area (Ai) and average internal perimeter (Pi) at both lung volumes and was found to be systematically higher at TLC by 13.5±9% on average, with the lower lobes displaying higher percent change in comparison to the lower lobes. The average internal diameter (Din) was evaluated to be 12.4±6.8% (MLV) and 10.8±6.3% (TLC) lower than the Dh, for all the examined bronchi, a result displaying statistical significance. Finally, the airway distensibility per bronchial segment and per generation was calculated to have an average value of 0.45±0.28, exhibiting high variability both between and within lung regions and generations. Mixed constriction/dilation patterns were recorded between the lung volumes, where a number of airways either failed to dilate or even constricted when observed at TLC. We conclude that the Dh is higher than Din, a fact that may have considerable effects on bronchial resistance or airway loss at proximal regions. Differences in caliber changes between lung regions are indicative of asthma-expression variability in the lung. However, airway distensibility at generation 3 seems to predict distensibility more distally.

## Introduction

Modeling of the lungs has become an important tool for both diagnostic [1–4] and research [5–11] purposes. Functional features of the lung such as airway compliance or distensibility [12–15], resistance [9,12,16–21], aerosol deposition [5,9,22–27] and ventilation distribution [10,19,26,28–32] computations are frequently made using a combination of medical images and the utilization of such models. Clearly, knowledge of the underlying lung morphologies is a key to these applications.

**Fig 1:**
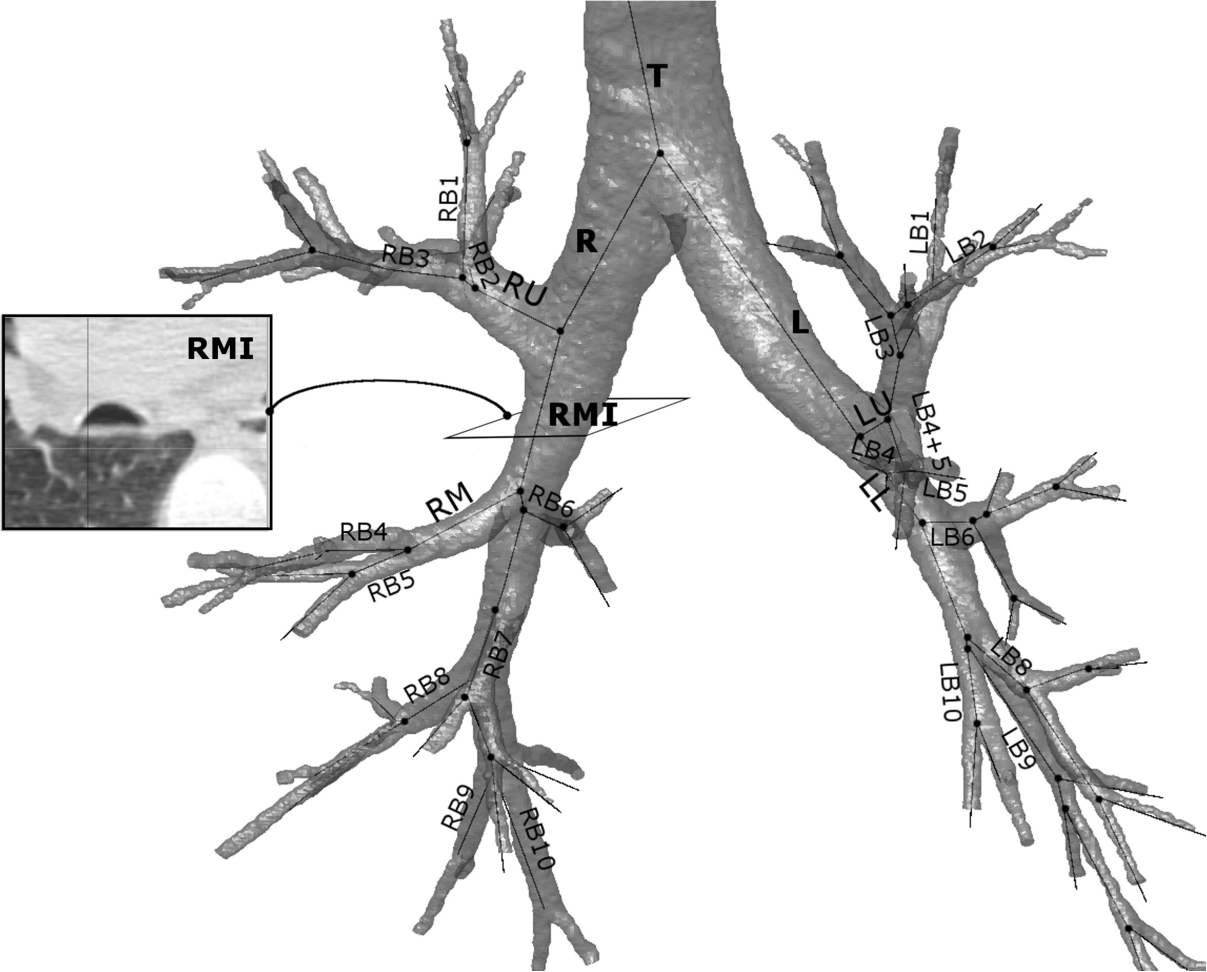
A segmented bronchial tree. The figure includes the central axis lines and the main airway names (down to the segmental level). The axial slice of the airway RMI is included on the left to demonstrate its non-circular cross-section.

In most cases the airway internal diameters (Din) [33–36], lumen areas (Ai) [33–35,37–39] or internal perimeters (Pi) [38,40] are evaluated. For computational models of gas-transport and aerosol deposition, idealized bronchial tree structures are developed and the above measurements used to define a network of bifurcating straight tubes [2,11,41,42]. However, it has been documented that this simplified models may not be accurate, particularly in the most proximal regions of the lung where the bronchi display transversal curvature [43,44]. Additionally, the shape of the cross-section of the airway is neither circular nor smooth [7,45] (Fig 1), a situation exacerbated in disease conditions [45–47]. For example, permanent airway remodeling in asthma causes the non-uniform swelling of the airway which, combined with increased mucous secretion, can form a complex lumen shape [45]. These effects need to be taken into account during functional modeling as they will clearly affect the flow.

A quantity that can be used to infer the potential functional influence of the internal shape of the airways is the Hydraulic Diameter (Dh), which is commonly used in fluid dynamics to characterize flow in non-circular channels [48]. Only a very few studies of this geometric feature have been performed in the past, with some concentrating in the extra-thoracic regions [49,50]. Recently, Choi et al. [38] have calculated regional Dh values and found that, when normalized to a predicted trachea diameter, Dh decreases in asthmatic bronchi when compared to healthy bronchi and correlates with certain Pulmonary Function Tests (PFTs).

In the current study, the influence of lung volume on the Dh, Ai and lumen internal perimeter Pi was examined. A set of bronchial measurements were performed in a mild intermittent or persistent group of asthma patients during methacholine (Mch)-induced broncho-constriction who were scanned with High Resolution Computed Tomography (HRCT) at two levels of lung inflation: mean lung volume (MLV) and total lung capacity (TLC). Subsequently, the Dh of individual airways was calculated using the measured average Ai and Pi and its correlation to the average Din was investigated. Finally, the distensibility of the airways was calculated and the correlation of lung volume change (ΔV/V) to lumen area change (ΔAi/Ai) was explored.

## Methods

### Medical image acquisition

The imaging protocol was completed at the Massachusetts General Hospital with IRB approval (Application No. 2007P000493) and is described in detail in [24]. In brief, the study included 24 volunteers diagnosed with mild intermittent or mild persistent asthma, as defined by the NIH Global Initiative for Asthma [51]. Written informed consent was provided by all volunteers. The first patient was recruited in December 2010. Demographic parameters are provided in Table 1. All subjects displayed reversible obstruction with inhaled albuterol. During an initial screening visit, the provocative concentration of MCh required to cause a 20% drop in the subject’s FEV1 (PC20) was estimated. On a second visit that same concentration was administered over 5 deep breaths by a DeVilbiss nebulizer and Rosenthal dosimeter (model 646, DeVilbiss Healthcare, Somerset, PA) with the subject already placed in a supine position within a PET/CT scanner (Biograph 64, Siemens AG).

**Table 1:** Patient demographic and lung function parameters (mean±std).

Gender	17F / 7M
Age (years)	19.8±1.7
Weight (kg)	66.8±10.6
Height (cm)	168.1±10.6
FEV_1_ (L)	3.8±0.8
FEV_1_ (% pred.)	103.2±10.4
FVC (L)	4.5±1.1
FVC (% pred.)	107.7±9.6
PC20	0.59±0.62
Right lung air volume (L) at MLV	1.294±0.441
Right lung air volume (L) at TLC	2.286±0.626
Left lung air volume (L) at MLV	1.136±0.374
Left lung air volume (L) at TLC	2.072±0.56

Two HRCT images were then obtained during breath hold; one at mean lung volume (MLV) and one at total lung capacity (TLC), respectively 5 and 30 minutes after the MCh challenge. The scanner was used in a helical mode to acquire 64 slices per rotation with a 0.6mm collimation. The imaging parameters were 120kvp, 80mA and a pitch of 1mm. The captured 3D images have slice thickness of 0.75mm with typical pixel spacing of 0.6x0.6 mm and slice separation of 0.5 mm.

### Bronchial geometry acquisition

The airway analysis was performed with the Pulmonary Workstation 2 (PW2) software (VIDA Diagnostics, Coralville, IA). The lungs, lobes and airway tree bronchi were segmented from the HRCT images down to the most distal distinguishable generation. A set of measurements was then performed on each segmented bronchus, including the average Din, Ai, WT, Pi and Po (outer perimeter). The manual and semi-automatic use of PW2 as a research tool is explained in detail in [36].

The airways in each segmented image were then uniquely identified, anatomically labeled and matched (Fig 1); only bronchi that were available at both levels of respiration were used. Standard nomenclature was used, matching the anatomical airway names with their respective acronyms [52]. Briefly, the airway names and their acronyms are the following: T (trachea), R (right main bronchus), L (left main bronchus), RMI (right intermediate), RU (right upper lobe), RM (right middle lobe), LU (left upper lobe) and LL (left lower lobe). The right lung segmental bronchi are for the right upper lobe RB1 (apical), RB2 (posterior), RB3 (anterior), for the right middle lobe RB4 (lateral) and RB5 (medial) and for the right lower lobe RB6 (superior), RB7 (medial basal), RB8 (anterior basal), RB9 (lateral basal), RB10 (posterior basal). For the left lung, the segmental bronchi in the upper lobe are LB1 (apical), LB2 (posterior), LB3 (anterior), LB4 (inferior), LB5 (superior) and in the lower lobe LB6 (superior), LB8 (anterior medial basal), LB9 (lateral basal) and LB10 (posterior basal).

The Dh was calculated according to the formula

$$Dh=\frac{4Ai}{Pi}$$ (1)

while airway distensibility was defined as

$$AD=\frac{\frac{\Delta Ai}{Ai}}{\sqrt[{3}]{{\left(\frac{\Delta V}{V}\right)}^{2}}}=\frac{\frac{{Ai}_{TLC}-{Ai}_{MLV}}{{Ai}_{MLV}}}{\sqrt[{3}]{{\left(\frac{V_{TLC}-V_{MLV}}{V_{MLV}}\right)}^{2}}},$$ (2)

where Ai_TLC_ and Ai_MLV_ is the airway lumen area at TLC and MLV respectively and V_TLC_ and V_MLV_ are the volumes of the bronchi’s subtended lungs (right or left depending on bronchus position). Paired Student t-tests were used to examine statistical significance between parameters (Dh vs Din) at both lung volumes (p<0.05). Linear regression analyses where assessed using the Pearson coefficient.

For practical purposes all lobar bronchi were assigned generation 2, segmental bronchi generation 3 etc. Given this definition, airway segmentation of matched airways was reliably available down to generation 5 only in the RB1, RB3 (right upper lobe) RB6, RB10 (right lower lobe) LB3 (left upper lobe) and LB10 (left lower lobe) segments.

## Results

The results from the Dh calculations are provided in Table 2 for both MLV and TLC groups. For convenience these results were organized per generation and per lobe. All bronchi displayed a 13.5±9% positive increase in Dh on average, ranging between 8.5±4.7% in the trachea and main bronchi and 19.6±9% in generations 4–5 of the left lower lobe. The lower lobes displayed higher % change on average than the upper lobes. In generation 3 that difference is 4.75% and 3.1% for the right and left lung respectively, while this trend persists in generations 4 (4.9% and 6.8%) but not fully in generation 5 (-0.7% and 6.2%). A decrease in Dh was also observed after full inspiration, but was rare (<5% of the examined samples). A Student t-test between the two groups revealed that, with the exception of RL lobe generations 2 and 5, all other comparisons were statistically significant (p<0.05).

**Table 2:** The hydraulic diameter per generation per lobe at MLV and TLC.

	Gen	Dh_MLV_		Dh_TLC_		% change		P	R^2^
		avg	std	avg	std	avg	std		
Trachea	0	13.48	1.98	14.61	2.07	7.7	4.6	<0.0001	0.876
Right	1	11.74	1.69	12.86	1.73	8.7	5.1	<0.0001	0.853
Left	1	9.09	1.58	10.12	1.7	10.1	5.2	<0.0001	0.883
RMI	2	8.68	1.22	9.4	1.22	7.7	4	<0.0001	0.895
RU	2	7.17	1.29	8.23	1.25	12.6	10.6	0.0001	0.469
RB1-3	3	3.82	0.8	4.47	0.9	14.4	7.6	<0.0001	0.848
	4	2.75	0.43	3.25	0.58	14.4	10.4	<0.0001	0.582
	5	2.55	0.41	2.91	0.37	10.8	13.8	0.0267	0.451
RM	2	5.11	0.79	5.75	0.75	11.1	7.3	<0.0001	0.718
RB4-5	3	3.37	0.44	3.85	0.69	11.7	7.7	0.0001	0.785
	4	2.53	0.4	2.89	0.47	12	9.8	0.0001	0.608
RL	2	8.12	1.57	8.37	1.57	2.8	10.7	0.2394	0.715
RB6-10	3	3.57	0.72	4.21	0.91	14.4	9.4	<0.0001	0.73
	4	2.57	0.5	3.09	0.65	16.1	9	<0.0001	0.712
	5	2.44	0.35	2.81	0.43	13.1	12.3	0.052	0.281
LU	2	7.84	1.33	8.55	1.21	8.3	6.7	<0.0001	0.801
LB1-5	3	3.24	0.6	3.73	0.64	12.9	8.9	0.0055	0.719
	4	2.84	0.47	2.83	0.61	12.8	8.7	0.0098	0.723
	5	2.69	0.36	3.16	0.6	13.3	13.2	0.0024	0.37
LL	2	7.34	0.86	8.45	1.02	13	5.7	<0.0001	0.706
LB6-10	3	3.92	0.65	4.73	0.82	16	10.2	<0.0001	0.592
	4	2.88	1.05	3.65	1.55	19.6	9	0.0001	0.647
	5	2.61	0.6	3.25	0.68	19.5	7	0.0012	0.881

Average and standard deviation values are given, along with percent change between the two lung volumes. The p value and correlation coefficient of a linear regression between the two groups is also provided.

**Fig 2:**
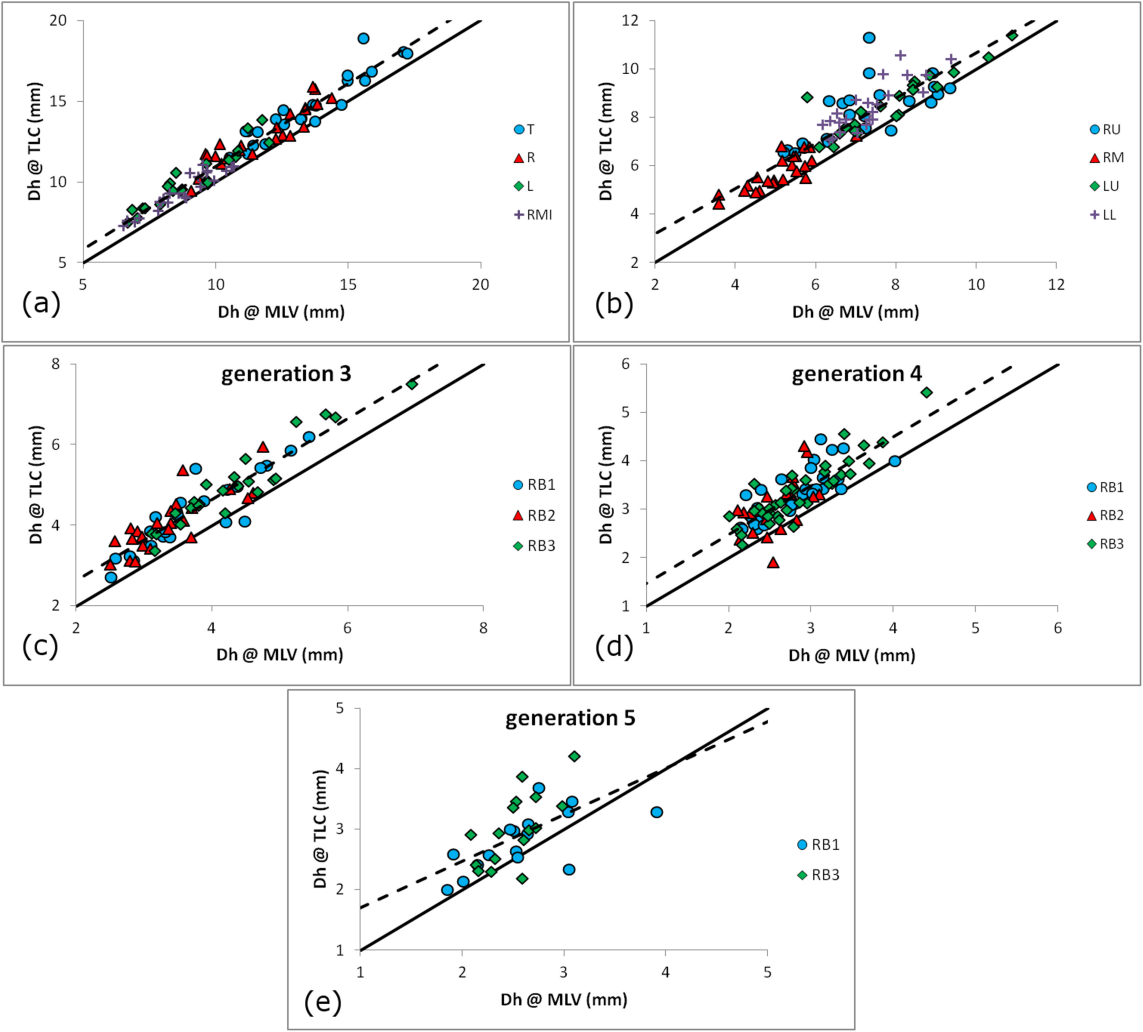
The hydraulic diameter MLV vs TLC. (a) The proximal airways, with T shown as blue circles ●, R as red triangles ▲, L as green diamonds ◆ and RMI as purple crosses **+**. (b) The lobar roots except RL, with RU shown as blue circles ●, RM as red triangles ▲, LU as green diamonds ◆ and LL as purple crosses **+**, (c) RU lobe generation 3, (d) RU lobe generation 4 and (e) RU lobe generation 5, with RB1 bronchi shown as blue circles ●, RB2 as red triangles ▲ and RB3 as green diamonds ◆. Airway naming terminology is explained in the Bronchial Geometry Acquisition section. The identity line (continuous line,––) and the combined data regression line (interrupted line,––) are also shown.

A linear regression analysis of Dh between MLV and TLC shows that the majority of the lobar regions display moderate or strong (R^2^>0.55) correlation to lung volume change (see Fig 2). In fact, the lowest correlation coefficients can be observed at generation 5 for the RU, RL and LU lobes, where measurement artifacts and more sparsely populated datasets are common due to diminishing airway sizes. The graphs of Fig 2 represent the effect of the two lung volumes on the Dh for the trachea, right and left main bronchi (Fig 2A), the lobar bronchi (Fig 2B), and the bronchi of generations 3–5 for the RU lobe (Fig 2C, 2D and 2E). With very few exceptions, the Dh increases for the large majority of the samples involved, with the trend lines almost parallel to the identity line, thus verifying the 13.5% average increase in Dh between lung volumes. These results were similar for all lobes.

**Fig 3:**
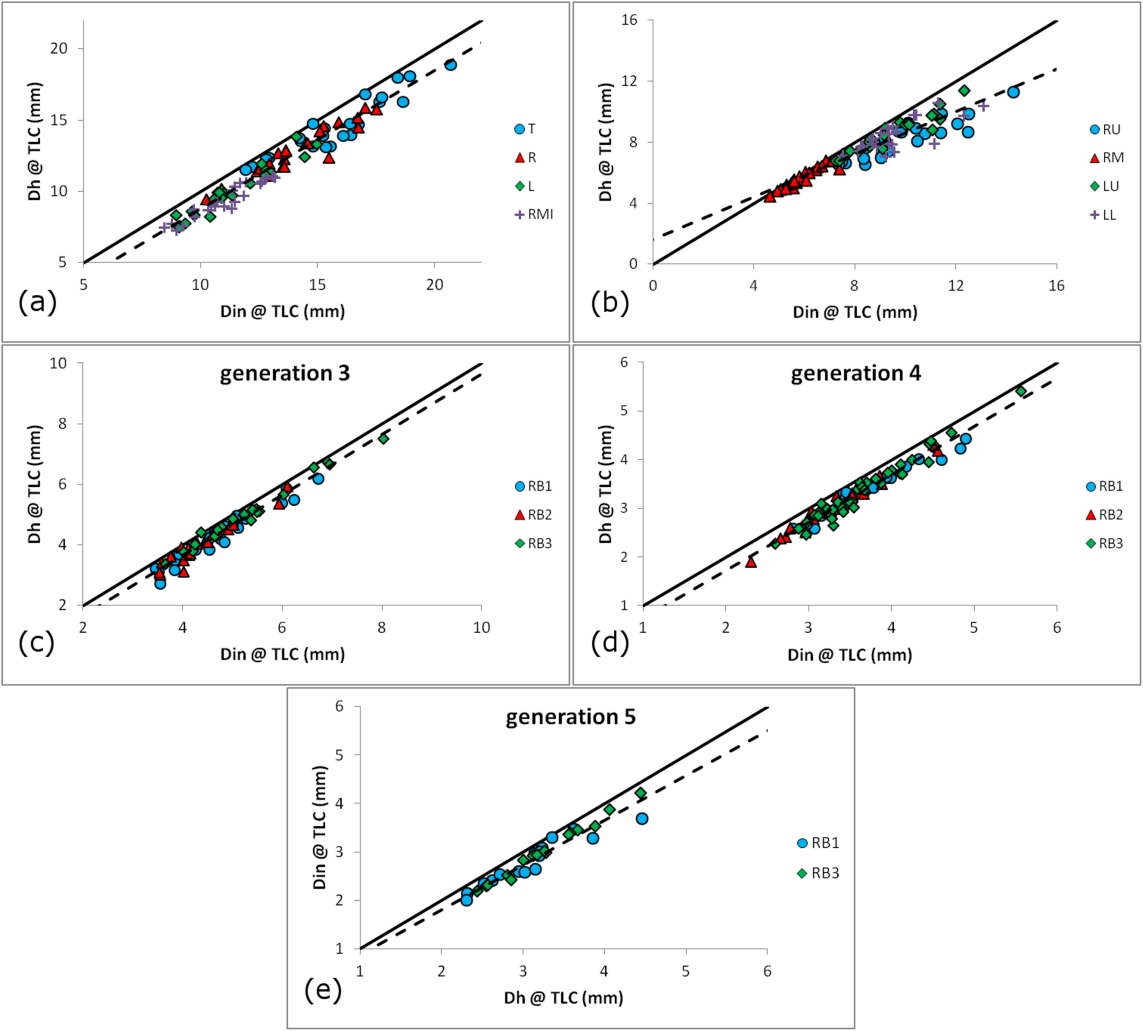
The hydraulic diameter versus average inner diameter. (a) The proximal airways, with T shown as blue circles ●, R as red triangles ▲, L as green diamonds ◆ and RMI as purple crosses **+**. (b) The lobar roots except RL, with RU shown as blue circles ●, RM as red triangles ▲, LU as green diamonds ◆ and LL as purple crosses **+**, (c) RU lobe generation 3, (d) RU lobe generation 4 and (e) RU lobe generation 5, with RB1 bronchi shown as blue circles ●, RB2 as red triangles ▲ and RB3 as green diamonds ◆. Airway naming terminology is explained in the Bronchial Geometry Acquisition section. The identity line (continuous line,––) and the combined data regression line (interrupted line,––) are also shown.

The comparison between Dh and Din, displayed in Fig 3, gives statistically significant results for all the airways involved in this study, with Din>Dh and very high correlation coefficients, as the R^2^ of the linear regression analyses are between 0.8734 (Fig 3B) and 0.9683 (Fig 3C). Very similar results could be observed for the other lobes for both MLV and TLC.

The average ratio Din/Dh was calculated to be 1.124±0.068 at MLV and 1.108±0.063 at TLC. Even though only 9 out of the 52 examined bronchi displayed statistically significant differences between the two lung volumes (significance given by p<0.01), almost all individual bronchial segments showed that the ratio Din/Dh is larger in MLV than in TLC.

The Ai and Pi were collected in Table 3 for all uniquely identifiable segmented bronchi in MLV and TLC (Ai in mm^2^ and Pi in mm). It can be seen that only 6 out of 19 possible generation 5 bronchial regions could be analyzed in the MLV images. For that reason, comparisons between the two lung volumes are only possible in these 5 particular regions. The lower lobes display higher change in airway caliber after inflation to TLC when compared to the upper lobes. For generation 3, the Ai change was 3.3% and 4.2% for the right and left lungs, respectively, while similar trends were found for generations 4 (6% and 13.4%) and 5 (4.9% and 12.2%).

**Table 3:** The measured lumen area (Ai) and inner perimeter (Pi) for specific airways at mean lung volume (MLV) and total lung capacity (TLC).

	Generations 0–2					Geneneration 3				
	Ai (mm^2^)		Pi (mm)			Ai (mm^2^)			Pi (mm)	
	MLV	TLC	MLV	TLC		MLV	TLC		MLV	TLC
T	162.2±47.36	181.43±51.55	47.8±7.1	49.44±7.1	RB1	12.87±7	16.66±6.78		13.27±4.41	14.65±2.96
R	120.5±32.35	141.17±36.57	40.47±5.56	43.4±5.77	RB2	10.1±3.74	14.23±5		11.49±2	13.6±2.31
L	72±24.26	88.06±30.25	30.8±5.42	33.78±6	RB3	16.02±7.16	21.1±8.98		14.31±2.95	16.4±3.29
RMI	66.86±17	77.83±18.66	30.25±3.88	32.53±4.1	RB4	8.51±2.67	11.7±6.64		10.73±1.72	12.26±3.02
RU	47.85±17.94	63.28±19.67	25.84±5.5	30.25±5.05	RB5	11.33±3.48	14.11±3.77		12.32±1.8	13.68±1.85
RM	21.76±6.5	27.3±7.14	16.75±2.52	18.77±2.54	RB6	21.55±22.47	29.52±29.47		17.26±9.09	20±10.09
LU	53.78±17.89	63.39±18.26	26.78±4.41	29.08±4.46	RB7	9.42±3.83	12.36±5.57		11.2±2.43	12.88±3.36
LL	47.87±12.7	62.37±15	25.53±3.99	29.16±3.74	RB8	10.41±2.72	14.91±4.41		11.8±1.45	14.23±2.24
LB4+5	21.64±9.97	28.01±9.6	16.58±4.09	18.89±3.56	RB9	8.09±1.86	11.9±4.21		10.41±1.24	12.64±2.64
LLR	28.59±9.29	40.5±12.43	19.1±2.91	22.56±3.39	RB10	11.36±3.47	16.06±5.9		12.28±1.77	14.43±2.29
					LB1	8.26±2.13	11.14±3.47		10.54±1.34	12.11±1.72
					LB2	5.8±2.07	7.71±2.95		8.78±1.45	10.11±1.77
					LB3	15.03±9.31	17.03±6.78		14.03±3.97	14.69±3.09
					LB4	9.23±2.67	11.26±4.14		11.19±1.47	12.23±2.32
					LB5	9.14±3.35	11.5±4.46		11.16±1.85	12.28±2.3
					LB6	13.26±3.65	23.3±8.55		13.33±1.77	17.7±3.42
					LB8	13.45±3.45	16.95±4.44		13.63±1.66	15.24±2.34
					LB9	12.47±4.68	16.3±5.75		13.1±2.23	14.8±3.32
					LB10	14.25±6.93	18.33±5.52		13.82±3.29	15.6±2.55
	Generation 4					Generation 5				
	Ai (mm^2^)		Pi (mm)			Ai (mm^2^)		Pi (mm)		
	MLV	TLC	MLV	TLC		MLV	TLC	MLV		TLC
RB1	6.88±2.11	8.74±3.02	9.61±1.38	10.77±1.75	RB1	6.04±2.58	6.54±2.03	8.99±1.84		9.13±1.8
RB2	5.93±1.39	7.84±2.79	9.05±1.1	10.11±1.65	RB2		6.22±2.04			8.91±1.87
RB3	7.11±2.57	9.44±3.53	9.71±1.56	11.04±1.86	RB3	5.55±1.16	6.98±2.63	8.71±0.84		9.39±2
RB4	5.3±1.49	6.82±2.29	8.61±1.17	9.43±1.66	RB4					
RB5	5.98±2.11	7.66±2.33	9.02±1.61	10.06±1.69	RB5		5.95±1.44			9.16±0.95
RB6	6.25±2.09	8.77±3.39	9.2±1.52	10.65±2.05	RB6	4.4±1.06	6.29±2.29	7.75±0.9		9.22±1.67
RB7	6.11±1.4	7.61±2.71	9.2±1.01	9.88±1.79	RB7					
RB8	5.81±3.53	8.3±4.95	8.77±2.27	10.39±2.69	RB8		7.54±1.73			9.99±1.71
RB9	4.71±1.9	6.61±2.25	7.9±1.55	9.27±1.51	RB9		4.99±1.02			8.37±0.87
RB10	6.67±3.16	9.44±4.54	9.31±2.25	10.98±2.85	RB10	5.99±1.68	8.12±3.81	8.94±1.19		10.22±2.33
LB1	6.88±1.8	7.48±2.61	9.79±1.28	9.84±1.69	LB1					
LB2		6.86±1.85		9.78±1.24	LB2					
LB3	2.5±3.15	9.42±4.21	14.03±3.97	10.9±2.38	LB3	6.43±1.61	7.69±2.86	9.44±1.2		10.12±1.78
LB4	6.66±1.73	7.56±2.23	9.43±1.2	9.85±1.68	LB4		5.76±1.37			8.02±2.36
LB5		7.8±1.47		10.36±0.92	LB5					
LB6	6.75±1.29	12.09±8.11	9.54±0.81	12.46±4.02	LB6		6.83±2.23			9.4±1.7
LB8	7.55±1.54	9.62±3.32	10.14±0.94	11.34±1.94	LB8		7.5±1.7			9.77±1.36
LB9	7.05±2.73	9.33±4.16	9.75±1.91	11.1±2.36	LB9		9.3±3.83			11.08±2.04
LB10	7.87±3.95	10.59±5.62	10.25±2.56	11.62±3.12	LB10	6.43±2.85	7.98±2.98	9.45±2.11		10.03±2.32

The results are given as mean±std values and only for segments where a sufficient number of samples (n≥8) is available.

**Fig 4:**
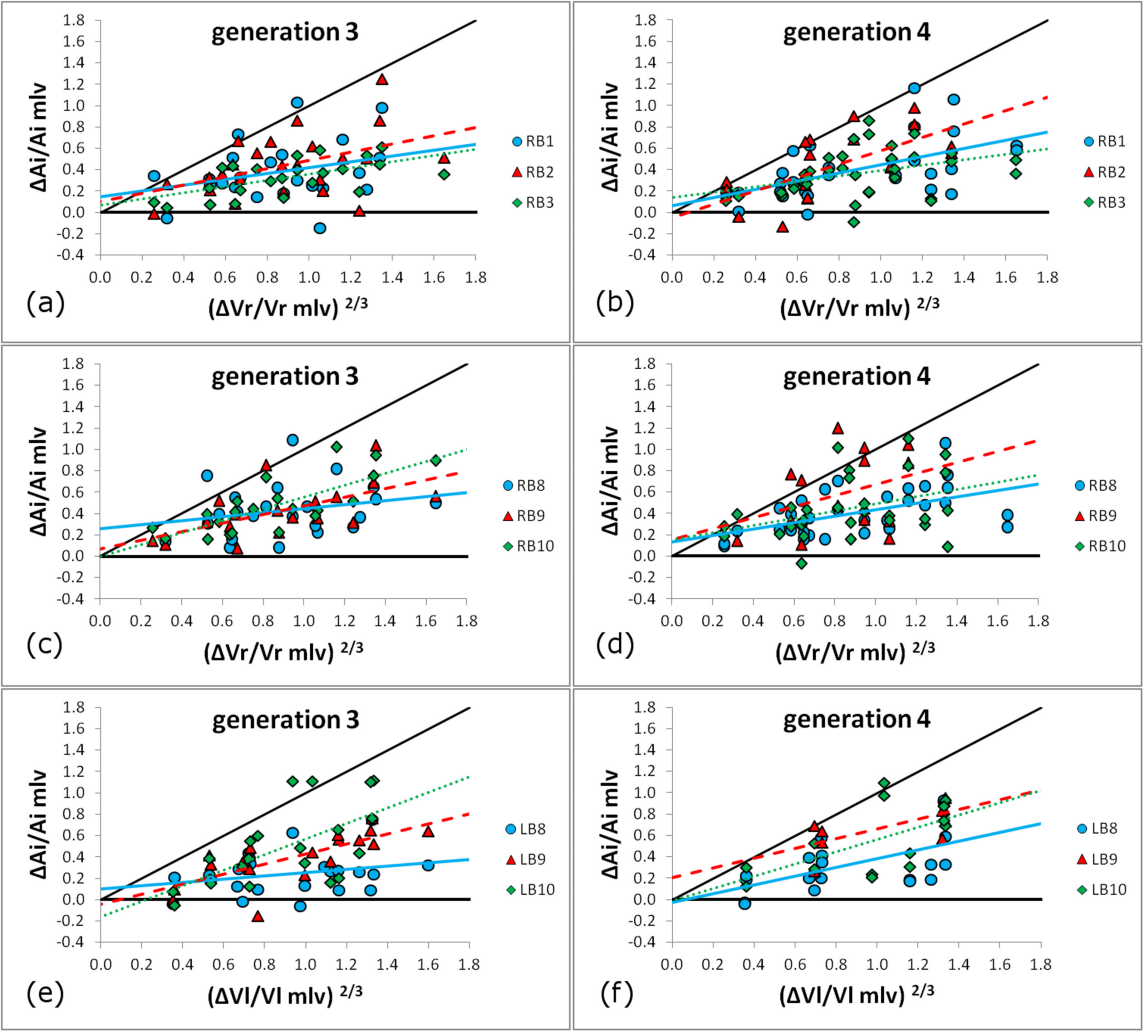
The influence of the change in lung volume between MLV and TLC on the lumen area. Data are shown for generations 3 and 4 for the right upper lobe (a and b), right lower lobe (c and d) and left lower lobe (e and f). (a, b) RB1 blue circle ●, RB2 red triangle ▲, RB3 green diamond ◆. (c, d) RB8 blue circle ●, RB9 red triangle ▲, RB10 green diamond ◆. (e, f) LB8 blue circle ●, LB9 red triangle ▲, LB10 green diamond ◆. The plotted regression lines are the following: RB1, RB8 and LB8 continuous line (blue––). RB2, RB9 and LB9 interrupted line (red––). RB3, RB10 and LB10 dotted line (green^….^). The identity line (diagonal black continuous line, black––) is also shown.

The average distensibility per airway, as defined in Eq (2), is recorded in Table 4. A box and whisker plot of these data for all available bronchi of generations 3 and 4 can be seen in the supplemental graph of S1 Fig. The distribution of the distensibility data in relation to the normal density function for generations 3 and 4 for the right and the left lung can be observed in the supplemental information graph S2 Fig., along with the relevant probability plots, shown in S3 Fig. A small number of data-points displayed distensibility values exceeding two standard deviations of the average value. These were considered outliers and removed from the dataset. The correlation between the lumen area change and lung volume change, as expressed in the same equation, was also examined and recorded in Table 4 in the form of the correlation coefficient R^2^. The plotted results of these correlations can be seen in Fig 4. The segmental airways of the right upper lobe (RB1-3, generation 3) display similar trends, with average values consistently below unity and close to 0.5, while the R^2^ values are low, indicating large value dispersions (Fig 4A). Equivalent trends could be observed for the same lung region for the generation 4 data (Fig 4B) with somewhat higher R^2^ values (R^2^_RB2-gen4_ = 0.4444).

**Table 4:** The distensibility and Pearson correlation coefficient between lung volume change and lumen area change, given per airway and generation.

	gen	Distensibility		R^2^		gen	Distensibility		R^2^
		avg.	std				avg.	Std	
RB1	3	0.41	0.34	0.0016	LB1	3	0.49	0.24	0.1498
	4	0.47	0.27	0.3198		4	0.12	0.17	0.9170
	5	0.32	0.19	0.0608					
RB2	3	0.51	0.29	0.2117	LB2	3	0.49	0.30	0.1691
	4	0.54	0.42	0.4444					
RB3	3	0.37	0.17	0.3620	LB3	3	0.32	0.23	0.4119
	4	0.43	0.21	0.1681		4	0.44	0.32	0.0628
	5	0.39	0.29	0.0500		5	0.33	0.33	0.7561
RB4	3	0.35	0.23	0.1098	LB4	3	0.36	0.31	0.5752
	4	0.44	0.27	0.2999		4	0.31	0.22	0.3183
RB5	3	0.28	0.15	0.4362	LB5	3	0.35	0.20	0.6899
	4	0.33	0.28	0.2910					
RB6	3	0.37	0.62	0.2261	LB6	3	0.83	0.31	0.326
	4	0.6	0.45	0.2129		4	0.91	0.51	0.5461
RB7	3	0.29	0.22	0.2289					
	4	0.26	0.14	0.3904					
RB8	3	0.51	0.32	0.0623	LB8	3	0.27	0.22	0.0710
	4	0.47	0.21	0.2720		4	0.37	0.22	0.3233
RB9	3	0.49	0.23	0.3592	LB9	3	0.51	0.33	0.6615
	4	0.71	0.41	0.1232		4	0.63	0.15	0.5838
RB10	3	0.57	0.22	0.5890	LB10	3	0.48	0.31	0.4362
	4	0.56	0.33	0.1485		4	0.52	0.24	0.4303
	5	0.39	0.31	0.7208		5	0.64	0.22	0.6476

Fig 4C and 4D show generation 3 and 4 data respectively for the right lower lobe. Airways RB8-10 show trends similar to the ones observed in the right upper lobe. An examination of generation 4 data indicates that, with the exception of airway RB10 (R^2^_RB10-gen3_ = 0.589), the change of lung volume does not correlate very well with the changes in lumen area. It can be observed that the distensibility of bronchus RB7 is consistently lower than that of RB6, 8, 9 and 10, even though statistical significance is only reached at generation 4 (p<0.05). The excessive variability of airway RB6 is also notable.

**Fig 5:**
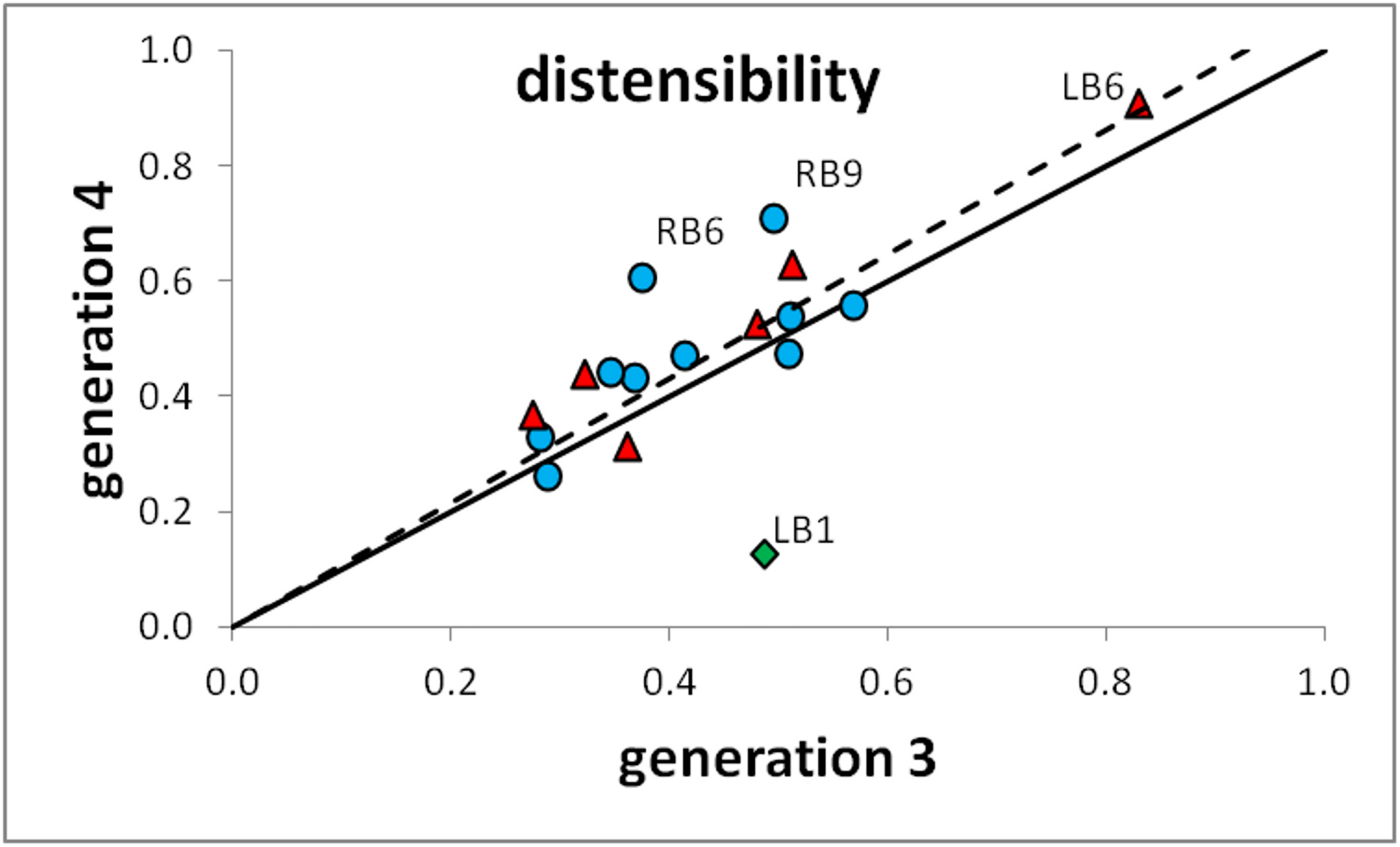
The average distensibility of individual region generation 3 and generation 4 airways. Right lung bronchial regions are plotted as blue circles ● while left lung regions are plotted as red triangles ▲. The outlier LB1 is represented as a green diamond ◆. The identity line (continuous line,––) and the combined data regression line (interrupted line,––) are also shown.

A lower dispersion of data can be observed in the left lung for all airways. Many analyzed bronchi, including LB3 (gen. 3 and 5), LB4, LB5, LB6 (gen. 4) LB9 (gen. 3 and 4) and LB10 (gen. 3, 4 and 5) had R^2^ > 0.4. Overall, the left lung had higher R^2^ values when equivalent regions from both lungs were compared. The influence of the left lung volume is distinct but relatively similar for bronchi LB8, LB9 and LB10 (Fig 4E). The generation 4 data can be seen in Fig 4F. Furthermore, statistical analysis revealed greater intra-region variability as compared to the right lung. For example, airway LB3 has lower distensibility (p<0.05) than both LB1 and LB2 in generation 3, while most comparisons between generation 4 bronchial distensibilities also gave statistically significant differences. As opposed to RB6, LB6 has lower variability and has the highest distensibility of all generation 3 airways of the left lung (p<0.05). Finally, similar levels of distensibility are observed between generations 3 and 4 in the right lung, while in the left lung statistical significance is reached only for airways LB1 (gen3>gen4, p = 0.0041) and LB3 (gen3<gen4, p = 0.0419). The Pearson correlation coefficient was found to be R^2^ = 0.4793 (full dataset) and R^2^ = 0.7401 (LB1 excluded). The distensibility of generation 3 vs generation 4 can be seen in Fig 5.

## Discussion

Even though several studies in the past have investigated the morphometry of the human bronchi during deep inspiration, only one study was found containing data on the Dh, calculated for airways at TLC and normalized to predicted trachea diameter [38]. If the TLC data of Table 2 are normalized to the value of the trachea for the purpose of comparison with Choi et al. [38], it is observed that our Dh values are within their overall range covering healthy, non severe and severe asthmatic patients, but do not strictly match the asthmatic range (S1 Table). Also, somewhat larger discrepancies appear in the left lung. This could be attributed to the known measurement artifacts in the left lung due to heart motion. Another explanation could be that the results of the Mch challenge administered to the patients in our study induce higher spatial variability within the lung in terms of broncho-constrictive effects.

Our results indicate that lower lobe bronchi change more in caliber than the upper lobe airways (Tables 2 and 3, Fig 4) at maximal lung inflation, especially in the left lung. In general, differences between lung regions have been reported in the literature for both healthy subjects [53] and COPD patients [54]. In the case of asthma, this difference may indicate that the effects of the methacholine challenge are more prominent in the upper regions of the lung. For example, Kotaru et al. [55], in a study examining the possible locations and patterns of airway response after different types of broncho-provocation on asthmatic subjects, did not report any regional differences but did find significant variability, especially with respect to the airway depth where the challenge is more effective. Furthermore, they observed complex morphometric alterations, not uniform nor graded, with mixed pattern dilation-constriction within and among anatomical regions. Another possibility is that the upper lobes of the human lung are more prone to disease, similar to COPD, as opposed to the lower lobes, the latter exhibiting more endurance during challenge but being faster to decline in function when more serious symptoms are observed [56]. This would be in line with the findings of [38], i.e. preferential regional occurrences of wall thickening in the upper lobes and airway narrowing in the lower lobes.

In addition to the above, larger diameter and luminal area changes between MLV and TLC exist in the more distal airways of the left lung (generations 4–5) rather than more proximally (generations 2–3, Tables 2 and 3). While investigating the effects of inspiration on airway dimensions of healthy subjects, Petersen et al. [53] pointed out that airways distend fully from generation 5 and deeper since their tissue is dominated by smooth muscle. Hoshino et al. [37] confirmed that there is an increasing correlation between Ai and clinical indices of disease as we penetrate deeper into the lung, confirming that airway remodeling takes place more distally. A similar study by Shimizu et al. [57] had comparable conclusions. Our results seem to partially agree with those of Brown et al. [58], who detected higher distensibilities (measured as %Ai change) in airways with diameter <3mm after Mch challenge. This could be attributed to the higher edema present in the smooth muscle, which may attenuate the radial traction produced by the inflation [59] or to airway remodeling of the extra-cellular matrix [45].

The high correlation coefficients and combined data regression lines of Fig 2 demonstrate a relatively mild and stable linear relationship for Dh between MLV and TLC, persistent throughout the lungs with some minor local variations. It is interesting to note that there is a relatively small number of bronchi displaying either no change or even a reduction of Dh at TLC (samples below the identity line). This dilation failure, or even the constriction, after inspiration at TLC might be statistically insignificant appositely to airway tree morphology but being located at proximal bronchi can have a considerable effect on symptoms [55,60]. Recently, Kim et al. [10] while investigating a coupled model of tissue deformation and network airflow observed that small airway constrictions can disturb flow patterns over large areas, reinforcing the findings of Venegas et al. [28,61] who showed that sudden closures of small airways break the system balance, leading to catastrophic shifts. They also found that the constriction in large airways that feed large volumes can profoundly affect lung function, an event that is not uncommon as we have discovered in this and a previous study by our group on asthmatic patients [36], where severe broncho constrictions were discovered as proximally as generation 3. The ventilation heterogeneity observed in asthma is therefore connected to regional anatomic heterogeneity, a fact confirmed by [7], making the use of average values over an entire generation misleading. Unfortunately, no method for the prediction of location and degree of proximal airway constrictions is currently available. Thus, morphometric models of disease that fail to give accurate representations of these defects, both geometrically and spatially, would result in functional models that could not exhibit important phenomena.

The comparison between the Dh and the Din demonstrated that the latter is almost uniformly larger than the former (Fig 3) by 12.5% at MLV and 10.8% at TLC, with the difference between lung volumes displaying statistical significance (p<0.01). In some airways, this difference can be considerably higher; for example, it was found to be 23±18% in the RB6 generation 3 airway. While these differences in diameter are relatively minor, functionally it should be remembered that for laminar and fully developed flow in a tube, the pressure drop is directly proportional to the flow rate but inversely proportional to the fourth power of diameter. Thus, a decrease in diameter by 10% would result in a 52% increase in pressure drop, all else being equal [48]. It is unclear if this systematic difference is a result of the measurement method used by the segmentation software or if it reflects a physical reality of the diseased human lung. If non-circular sections are considered throughout the segmented airway tree, as is implied by our results and also by [18,38], finding the Dh should be a priority over the measurement of Din for reasons concerning both airway shape and overall airflow resistance.

Since airway resistance depends on the Dh [48], the effect of this difference has a multiplicative effect on overall tree resistance calculations as the number of airways doubles with each successive generation distally into the lung [62]. Furthermore, even though the Din/Dh displayed statistically significant differences between MLV and TLC in only 9 out of 52 examined segments, the ratio was found to be consistently larger at MLV, indicating a trend for higher airway resistance at this breathing condition. The analysis of HRCT, due to the limited spatial resolution, was not sufficient to reveal the full complexity of the bronchial perimeter shape, providing an elliptical cross-section approximation. For that reason, the difference in the Din/Dh ratio between the two lung volume states probably represents a small increase in airway circularity at TLC; i.e., there is an anisotropic airway expansion across its circumference during inspiration. This is probably related to the elastic properties of the lung parenchyma, which is an important feature of the mechanics of breathing in disease conditions [10,63].

Several previous studies have reported the bronchial lumen area, either directly or as percentage of total area, for various uniquely identified airways and for various states of health and disease. The results were mostly presented either as average values per airway generation [38,64] or are given only for specific, easily identifiable generation 3 airways that may act as surrogate markers of lung function and tree morphology [34,54,65–67], or both [37,53,57]. In this study, Ai and Pi measurements were provided in Table 3 for all segmented bronchi for generations 3–5 for both MLV and TLC and without any normalization. This was done mostly because, as mentioned earlier, concentrating on specific airways may ignore localized characteristics of disease or disease progression due to the intense intra-subject variability of asthma. Furthermore, as Bakker et al. [54] suggested, bronchial measurements should not be presented as an average value for the whole lung, a statement supported by the findings of Fain and coworkers [68] who found that normalization of airway measures did not eliminate dependence on airway segment and other patient specific factors in a group of normal and asthmatic subjects. Finally, individualized airway morphometric information can be really useful for lung modeling applications [11,21]

Many techniques have been used in the past to determine what is the influence of lung inflation, if any, on the airway geometry [13,54,58,69]. In this study, a definition of airway distensibility that explores relative changes in Ai and ΔV was adopted. The results are reported for all bronchial segments in Table 4. The influence of volume change per lung on Ai change was also reported as the Pearson correlation coefficient and is displayed in the graphs of Fig 4. These data suggest that volume changes in the right lung only moderately affect the Ai in the relevant airways, while better correlations exist when the left lung is considered. This might be due to the fact that the morphometric measurements conducted in this study where made on static images (HRCT) while the airway distensibility has been shown to be primarily affected by the dynamic ability to change lung volumes in asthma [70].

The formulas of distensibility suggested by Bakker et al. [54] and Diaz et al. [35] were also examined. Even though the best correlations between ΔAi and ΔV were observed with the definition adopted by [35], we believe that the definition of Eq (2) makes the assessment of bronchial distensibility easier both within and between different airways and different generations. This is because, if the value of distensibility in Eq (2) is unity, one can claim that the change in area is the same as the change in lung parenchyma, i.e. the airway and the parenchyma display similar compliance. In the group of patients analyzed in this study, the distensibility was calculated almost unilaterally below unity, with an average of 0.45±0.28, displaying variation between different lung regions. This failure of the central airway lumen to dilate to an equivalent degree with the inflation of the lung should be expected due to the tissue composition of these bronchi. For example, [53] considers that full airway distensibility is only achievable after generation 5 in healthy lungs, where soft tissue is dominant. However, in asthma extra bronchial rigidity might be expected due to mechanical influence of the bronchial wall edema,. Furthermore, airway wall contractility should also be affected by the increased tone effected by the methacholine challenge. Unfortunately, no healthy subjects were included in this study for comparison purposes. It has to be noted that if the effects of respiration and lung volume-change on airway size are to be comparable across studies, similar definitions must be universally adopted.

In the context of mathematical modeling of the bronchial tree, the results provided in this paper can potentially be valuable, especially when parent-daughter relationships are used to calculate bronchial geometry information of distal lung regions [2,8,11,41]. The high correlation between Din and Dh allows for a straightforward linear conversion while the data of Tables 2, 3 and 4 provide sufficient information on Dh, Ai and Pi for the relevant adjustments to be made to the models, depending on respiratory conditions, initial information and other system requirements for the modeling application. Many modern tracheo-bronchial tree models utilize medical images of the lung acquired at TLC to obtain the initial conditions for airway tree propagation to the acinar level [11,21,71]. These models, in turn, are used to simulate conditions at regular breathing without the geometric (circularity, resistance, non-persistent stenoses) and ventilation discrepancies being taken into account. This misconception has recently been recognized by researchers using such models of the lung. For example, in a CFD study on aerosol deposition in bronchial tree models extracted from HRCT images at MLV and TLC, Katz et al. [27] concluded that lung volume should be explicitly considered when using deposition models. Bordas et al. [21], lacking relevant information for a population of asthmatic patients, used the minimum inner diameters of the segmented airways in conjunction with a circular cross-section assumption to predict airflow in the lung. Another method was used by Kim et al. [10], who implemented their dynamic ventilation model only after uniformly scaling down the bronchial tree model obtained at TLC by a scale factor $\sqrt[{3}]{FRC/TLC}$.

One limitation of this study is the lack of sufficient resolution in the medical imaging modality used to assess the real shape of airway internal cross-section perimeter. Especially in disease, this perimeter might have a complex geometry due to swelling, hyperplasia of goblet cells, mucous excretions etc. [72] which, in turn, could considerably affect airflow and particle deposition. Therefore, these changes in circularity between MLV and TLC that are reported here could be connected to the “unfolding” of the bronchus as the pressure changes in the lung drive the airways to obtain higher volumes. Furthermore, comparisons between MLV and TLC could only concentrate on airways that could be segmented in both lung volumes. This undoubtedly biases the data in favor of larger airways, especially in more distal lung regions where image resolution is not sufficient for airway segmentation at MLV. Finally, as stated earlier, no dynamic effects of respiration could be taken into account as there is no temporal component in the medical images used herein.

In conclusion, this study was concerned with the examination of the hydraulic diameter human bronchial tree as calculated from the lumen area and inner perimeter at both mean lung volume and total lung capacity. The relationships between several morphometric features were investigated, along with the effects of lung volume change on airway shape. The airway hydraulic diameter was found to be consistently higher than average inner diameter at both investigated lung volumes, a fact that can have considerable effects on resistance and pressure loss at proximal lung regions. Furthermore, differences in caliber changes were observed between upper and lower lobe bronchi, which might be indicative of differences in asthma expression in different lung regions. A failure of some airways to dilate at TLC was also documented, confirming the mixed constriction-dilation patterns of asthmatic airways that lead to the patchiness of lung ventilation characteristic of this disease. Finally, generation 3 distensibility was predictive of distensibility at generation 4. These results could be used for adapting models of the human trachea-bronchial tree and the interpretation of results for ventilation and aerosol deposition simulations.

## Supporting information

S1 Fig Distensibility box and whisker plot. A box-plot of the distensibility for all the available airways of generations 3 and 4. Outliers are observed at above and under 1.5 Inter-Quartile range.
(TIF)

S2 Fig Distensibility data distribution. This is a histogram of the distribution of the distensibility data of the right and left lung for the airways of generation 3 and 4. The best-fit normal density function is also shown as a red line.
(TIF)

S3 Fig Distensibility probability plot. The probability plot comparing the distensibility data to the normal distribution (reference line—-), created for the right and left lung generation 3 and 4 data. It can be observed that the fit of the data to the reference line deteriorates when distensibility values exceed one.
(TIF)

S1 Table Normalized hydraulic diameter data. The hydraulic diameter data are normalized to the hydraulic diameter calculated for the trachea and are collected for the same airway tree bronchial regions as the ones considered by Choi et al. [38] (where available). The data are give as average values ± standard deviation values.
(DOCX)

S1 File Data processing file. This is the excel file used to gather and statistically analyze the bronchial tree morphometric information that are investigated in this study. The file contains the average inner diameter, average lumen area, average inner perimeter, calculated hydraulic diameter and several definitions for airway distensibility. The data are gathered in a variety of ways including per individually identified airway and per generation. The file also contains statistical analysis.
(ZIP)

## References

[1] ICRP, Protection IC on R. ICRP Publication 66: Human Respiratory Tract Model for Radiological Protection. Elsevier Health Sciences; 1994.

[2] Martonen TB, Yang Y, Hwang D, Fleming JS. Computer model of human lung morphology to complement SPECT analyses. Int J Biomed Comput. 1995;40: 5–16. 8557404 10.1016/0020-7101(95)01106-o

[3] Liang L, Larsen EW, Chetty IJ. An anatomically realistic lung model for Monte Carlo-based dose calculations. Med Phys. 2007;34: 1013–1025. doi: 10.1118/1.2437284 17441248 10.1118/1.2437284

[4] Rosati Rowe JA, Burton R, McGregor G, McCauley R, Tang W, Spencer R. Development of a three-dimensional model of the human respiratory system for dosimetric use. Theor Biol Med Model. 2013;10: 28 doi: 10.1186/1742-4682-10-28 23634755 10.1186/1742-4682-10-28 PMC3691747

[5] Fleming J s., Nassim M, Hashish A h., Bailey A g., Conway J, Holgate S, et al Description of Pulmonary Deposition of Radiolabeled Aerosol by Airway Generation Using a Conceptual Three Dimensional Model of Lung Morphology. J Aerosol Med. 1995;8: 341–356. doi: 10.1089/jam.1995.8.341

[6] Tgavalekos NT, Venegas JG, Suki B, Lutchen KR. Relation between structure, function, and imaging in a three-dimensional model of the lung. Ann Biomed Eng. 2003;31: 363–373. 12723678 10.1114/1.1557972

[7] Vial L, Perchet D, Fodil R, Caillibotte G, Fetita C, Prêteux F, et al Airflow modeling of steady inspiration in two realistic proximal airway trees reconstructed from human thoracic tomodensitometric images. Comput Methods Biomech Biomed Engin. 2005;8: 267–277. doi: 10.1080/10255840512331389280 16298849 10.1080/10255840512331389280

[8] Florens M, Sapoval B, Filoche M. An anatomical and functional model of the human tracheobronchial tree. J Appl Physiol Bethesda Md 1985. 2011;110: 756–763. doi: 10.1152/japplphysiol.00984.2010 21183626 10.1152/japplphysiol.00984.2010

[9] Katz I, Pichelin M, Montesantos S, Majoral C, Martin A, Conway J, et al Using Helium-Oxygen to Improve Regional Deposition of Inhaled Particles: Mechanical Principles. J Aerosol Med Pulm Drug Deliv. 2014;27: 71–80. doi: 10.1089/jamp.2013.1072 24383961 10.1089/jamp.2013.1072

[10] Kim M, Bordas R, Vos W, Hartley RA, Brightling CE, Kay D, et al Dynamic flow characteristics in normal and asthmatic lungs. Int J Numer Methods Biomed Eng. 2015;31 doi: 10.1002/cnm.2730 26033976 10.1002/cnm.2730

[11] Montesantos S, Katz I, Pichelin M, Caillibotte G. The Creation and Statistical Evaluation of a Deterministic Model of the Human Bronchial Tree from HRCT Images. PLOS ONE. 2016;11: e0168026 doi: 10.1371/journal.pone.0168026 27977730 10.1371/journal.pone.0168026 PMC5157997

[12] Brown NJ, Salome CM, Berend N, Thorpe CW, King GG. Airway distensibility in adults with asthma and healthy adults, measured by forced oscillation technique. Am J Respir Crit Care Med. 2007;176: 129–137. doi: 10.1164/rccm.200609-1317OC 17463413 10.1164/rccm.200609-1317OC

[13] Kermode JA, Brown NJ, Hardaker KM, Farah CS, Berend N, King GG, et al The effect of airway remodelling on airway hyper-responsiveness in asthma. Respir Med. 2011;105: 1798–1804. doi: 10.1016/j.rmed.2011.07.010 21820298 10.1016/j.rmed.2011.07.010

[14] Kelly VJ, Brown NJ, Sands SA, Borg BM, King GG, Thompson BR. Effect of airway smooth muscle tone on airway distensibility measured by the forced oscillation technique in adults with asthma. J Appl Physiol Bethesda Md 1985. 2012;112: 1494–1503. doi: 10.1152/japplphysiol.01259.2011 22362406 10.1152/japplphysiol.01259.2011

[15] Brown RH, Togias A. Measurement of Intra-individual Airway Tone Heterogeneity and its Importance in Asthma. J Appl Physiol Bethesda Md 1985. 2016; jap.00545.2015. doi: 10.1152/japplphysiol.00545.2015 27103654 10.1152/japplphysiol.00545.2015 PMC4967252

[16] Bokov P, Mauroy B, Revel M-P, Brun P-A, Peiffer C, Daniel C, et al Lumen areas and homothety factor influence airway resistance in COPD. Respir Physiol Neurobiol. 2010;173: 1–10. doi: 10.1016/j.resp.2010.05.011 20478416 10.1016/j.resp.2010.05.011

[17] Katz IM, Martin AR, Muller P-A, Terzibachi K, Feng C-H, Caillibotte G, et al The ventilation distribution of helium–oxygen mixtures and the role of inertial losses in the presence of heterogeneous airway obstructions. J Biomech. 2011;44: 1137–1143. doi: 10.1016/j.jbiomech.2011.01.022 21316683 10.1016/j.jbiomech.2011.01.022

[18] Wongviriyawong C, Harris RS, Zheng H, Kone M, Winkler T, Venegas JG. Functional effect of longitudinal heterogeneity in constricted airways before and after lung expansion. J Appl Physiol Bethesda Md 1985. 2012;112: 237–245. doi: 10.1152/japplphysiol.01400.2010 21940845 10.1152/japplphysiol.01400.2010 PMC3290421

[19] Wongviriyawong C, Harris RS, Greenblatt E, Winkler T, Venegas JG. Peripheral resistance: a link between global airflow obstruction and regional ventilation distribution. J Appl Physiol Bethesda Md 1985. 2013;114: 504–514. doi: 10.1152/japplphysiol.00273.2012 23123354 10.1152/japplphysiol.00273.2012 PMC3569839

[20] Borojeni AAT, Noga ML, Martin AR, Finlay WH. Validation of airway resistance models for predicting pressure loss through anatomically realistic conducting airway replicas of adults and children. J Biomech. 2015;48: 1988–1996. doi: 10.1016/j.jbiomech.2015.03.035 25912661 10.1016/j.jbiomech.2015.03.035

[21] Bordas R, Lefevre C, Veeckmans B, Pitt-Francis J, Fetita C, Brightling CE, et al Development and Analysis of Patient-Based Complete Conducting Airways Models. Sznitman J, editor. PLOS ONE. 2015;10: e0144105 doi: 10.1371/journal.pone.0144105 26656288 10.1371/journal.pone.0144105 PMC4684353

[22] Koblinger L, Hofmann W. Monte Carlo modeling of aerosol deposition in human lungs. Part I: Simulation of particle transport in a stochastic lung structure. J Aerosol Sci. 1990;21: 661–674. doi: 10.1016/0021-8502(90)90121-D

[23] Lee DY, Lee JW. Dispersion of aerosol bolus during one respiration cycle in a model of lung airways. J Aerosol Sci. 2002;33: 1219–1234. doi: 10.1016/S0021-8502(02)00053-8

[24] Greenblatt EE, Winkler T, Harris RS, Kelly VJ, Kone M, Katz I, et al What Causes Uneven Aerosol Deposition in the Bronchoconstricted Lung? A Quantitative Imaging Study. J Aerosol Med Pulm Drug Deliv. 2015; doi: 10.1089/jamp.2014.1197 25977979 10.1089/jamp.2014.1197 PMC4770852

[25] Tian G, Hindle M, Lee S, Longest PW. Validating CFD Predictions of Pharmaceutical Aerosol Deposition with In Vivo Data. Pharm Res. 2015;32: 3170–3187. doi: 10.1007/s11095-015-1695-1 25944585 10.1007/s11095-015-1695-1 PMC4580521

[26] Greenblatt EE, Winkler T, Harris RS, Kelly VJ, Kone M, Katz I, et al Regional Ventilation and Aerosol Deposition with Helium-Oxygen in Bronchoconstricted Asthmatic Lungs. J Aerosol Med Pulm Drug Deliv. 2016;29: 260–272. doi: 10.1089/jamp.2014.1204 26824777 10.1089/jamp.2014.1204 PMC4894015

[27] Katz I, Pichelin M, Montesantos S, Murdock A, Fromont S, Venegas J, et al The Influence of Lung Volume during Imaging on CFD within Realistic Airway Models. Aerosol Sci Technol. 2016;0: 00–00. doi: 10.1080/02786826.2016.1254721

[28] Venegas JG, Schroeder T, Harris S, Winkler RT, Melo MFV. The distribution of ventilation during bronchoconstriction is patchy and bimodal: a PET imaging study. Respir Physiol Neurobiol. 2005;148: 57–64. doi: 10.1016/j.resp.2005.05.023 15994134 10.1016/j.resp.2005.05.023

[29] Grebenkov DS, Guillot G, Sapoval B. Restricted diffusion in a model acinar labyrinth by NMR: theoretical and numerical results. J Magn Reson San Diego Calif 1997. 2007;184: 143–156. doi: 10.1016/j.jmr.2006.09.026 17055758 10.1016/j.jmr.2006.09.026

[30] Venegas J. Linking ventilation heterogeneity and airway hyperresponsiveness in asthma. Thorax. 2007;62: 653–654. doi: 10.1136/thx.2006.073239 17687092 10.1136/thx.2006.073239 PMC2117263

[31] Swan AJ, Clark AR, Tawhai MH. A computational model of the topographic distribution of ventilation in healthy human lungs. J Theor Biol. 2012;300: 222–231. doi: 10.1016/j.jtbi.2012.01.042 22326472 10.1016/j.jtbi.2012.01.042 PMC3308631

[32] Yin Y, Choi J, Hoffman EA, Tawhai MH, Lin C-L. A multiscale MDCT image-based breathing lung model with time-varying regional ventilation. J Comput Phys. 2013;244: 168–192. doi: 10.1016/j.jcp.2012.12.007 23794749 10.1016/j.jcp.2012.12.007 PMC3685439

[33] Montaudon M, Desbarats P, Berger P, de Dietrich G, Marthan R, Laurent F. Assessment of bronchial wall thickness and lumen diameter in human adults using multi-detector computed tomography: comparison with theoretical models. J Anat. 2007;211: 579–588. doi: 10.1111/j.1469-7580.2007.00811.x 17919291 10.1111/j.1469-7580.2007.00811.x PMC2375785

[34] Chae EJ, Kim T-B, Cho YS, Park C-S, Seo JB, Kim N, et al Airway Measurement for Airway Remodeling Defined by Post-Bronchodilator FEV1/FVC in Asthma: Investigation Using Inspiration-Expiration Computed Tomography. Allergy Asthma Immunol Res. 2011;3: 111–117. doi: 10.4168/aair.2011.3.2.111 21461250 10.4168/aair.2011.3.2.111 PMC3062789

[35] Diaz AA, Come CE, Ross JC, San José Estépar R, Han MK, Loring SH, et al Association between airway caliber changes with lung inflation and emphysema assessed by volumetric CT scan in subjects with COPD. Chest. 2012;141: 736–744. doi: 10.1378/chest.11-1026 21940776 10.1378/chest.11-1026 PMC3296457

[36] Montesantos S, Katz I, Fleming J, Majoral C, Pichelin M, Dubau C, et al Airway Morphology From High Resolution Computed Tomography in Healthy Subjects and Patients With Moderate Persistent Asthma. Anat Rec. 2013;296: 852–866. doi: 10.1002/ar.22695 23564729 10.1002/ar.22695

[37] Hoshino M, Matsuoka S, Handa H, Miyazawa T, Yagihashi K. Correlation between airflow limitation and airway dimensions assessed by multidetector CT in asthma. Respir Med. 2010;104: 794–800. doi: 10.1016/j.rmed.2009.12.005 20053544 10.1016/j.rmed.2009.12.005

[38] Choi S, Hoffman EA, Wenzel SE, Castro M, Fain SB, Jarjour NN, et al Quantitative assessment of multiscale structural and functional alterations in asthmatic populations. J Appl Physiol Bethesda Md 1985. 2015;118: 1286–1298. doi: 10.1152/japplphysiol.01094.2014 25814641 10.1152/japplphysiol.01094.2014 PMC4436982

[39] Hartley RA, Barker BL, Newby C, Pakkal M, Baldi S, Kajekar R, et al Relationship between lung function and quantitative computed tomographic parameters of airway remodeling, air trapping, and emphysema in patients with asthma and chronic obstructive pulmonary disease: A single-center study. J Allergy Clin Immunol. 2016;137: 1413–1422.e12. doi: 10.1016/j.jaci.2016.02.001 27006248 10.1016/j.jaci.2016.02.001 PMC4852952

[40] Donohue KM, Hoffman EA, Baumhauer H, Guo J, Ahmed FS, Lovasi GS, et al Asthma and lung structure on computed tomography: the Multi-Ethnic Study of Atherosclerosis Lung Study. J Allergy Clin Immunol. 2013;131: 361–368.e1–11. doi: 10.1016/j.jaci.2012.11.036 23374265 10.1016/j.jaci.2012.11.036 PMC3564253

[41] Kitaoka H, Takaki R, Suki B. A three-dimensional model of the human airway tree. J Appl Physiol Bethesda Md 1985. 1999;87: 2207–2217. 10.1152/jappl.1999.87.6.2207 10601169

[42] Tawhai M, Pullan AJ, Hunter PJ. Generation of an anatomically based three-dimensional model of the conducting airways. Ann Biomed Eng. 2000;28: 793–802. 11016416 10.1114/1.1289457

[43] Sauret V, Goatman KA, Fleming JS, Bailey AG. Semi-automated tabulation of the 3D topology and morphology of branching networks using CT: application to the airway tree. Phys Med Biol. 1999;44: 1625–1638. 10442701 10.1088/0031-9155/44/7/304

[44] Tawhai MH, Burrowes KS. Developing integrative computational models of pulmonary structure. Anat Rec B New Anat. 2003;275: 207–218. doi: 10.1002/ar.b.10034 14628321 10.1002/ar.b.10034

[45] Eskandari M, Kuschner WG, Kuhl E. Patient-Specific Airway Wall Remodeling in Chronic Lung Disease. Ann Biomed Eng. 2015; 1–14. 25821112 10.1007/s10439-015-1306-7 PMC4573354

[46] Busse WW, Banks-Schlegel S, Wenzel SE. Pathophysiology of severe asthma. J Allergy Clin Immunol. 2000;106: 1033–1042. doi: 10.1067/mai.2000.111307 11112883 10.1067/mai.2000.111307

[47] Hogg JC. Pathophysiology of airflow limitation in chronic obstructive pulmonary disease. The Lancet. 2004;364: 709–721. 10.1016/S0140-6736(04)16900-6 15325838

[48] Shaughnessy EJ, Katz IM, Schaffer JP. Introduction to fluid mechanics [Internet]. Oxford University Press New York; 2005 Available: http://www.academia.edu/download/43976514/Introduction_to_Fluid_Mechanics.pdf

[49] Rembold CM, Suratt PM. Airway turbulence and changes in upper airway hydraulic diameter can be estimated from the intensity of high frequency inspiratory sounds in sleeping adults. J Physiol. 2014;592: 3831–3839. doi: 10.1113/jphysiol.2014.272302 24973405 10.1113/jphysiol.2014.272302 PMC4192706

[50] Walenga RL, Tian G, Hindle M, Yelverton J, Dodson K, Longest PW. Variability in Nose-to-Lung Aerosol Delivery. J Aerosol Sci. 2014;78: 11–29. doi: 10.1016/j.jaerosci.2014.08.003 25308992 10.1016/j.jaerosci.2014.08.003 PMC4187112

[51] Bateman ED, Hurd SS, Barnes PJ, Bousquet J, Drazen JM, FitzGerald M, et al Global strategy for asthma management and prevention: GINA executive summary. Eur Respir J. 2008;31: 143–178. doi: 10.1183/09031936.00138707 18166595 10.1183/09031936.00138707

[52] Holinger P. The nomenclature of broncho-pulmonary anatomy. Thorax. 1950;5: 222. 14776714 10.1136/thx.5.3.222 PMC1018336

[53] Petersen J, Wille MMW, Rakêt LL, Feragen A, Pedersen JH, Nielsen M, et al Effect of inspiration on airway dimensions measured in maximal inspiration CT images of subjects without airflow limitation. Eur Radiol. 2014;24: 2319–2325. doi: 10.1007/s00330-014-3261-3 24903230 10.1007/s00330-014-3261-3

[54] Bakker ME, Stolk J, Reiber JHC, Stoel BC. Influence of inspiration level on bronchial lumen measurements with computed tomography. Respir Med. 2012;106: 677–686. doi: 10.1016/j.rmed.2011.11.013 22154247 10.1016/j.rmed.2011.11.013

[55] Kotaru C, Coreno A, Skowronski M, Muswick G, Gilkeson RC, McFadden ER. Morphometric changes after thermal and methacholine bronchoprovocations. J Appl Physiol Bethesda Md 1985. 2005;98: 1028–1036. doi: 10.1152/japplphysiol.01186.2003 15542566 10.1152/japplphysiol.01186.2003

[56] Kundu S, Gu S, Leader JK, Tedrow JR, Sciurba FC, Gur D, et al Assessment of lung volume collapsibility in chronic obstructive lung disease patients using CT. Eur Radiol. 2013;23: 1564–1572. doi: 10.1007/s00330-012-2746-1 23494492 10.1007/s00330-012-2746-1 PMC3657332

[57] Shimizu K, Hasegawa M, Makita H, Nasuhara Y, Konno S, Nishimura M. Airflow limitation and airway dimensions assessed per bronchial generation in older asthmatics. Respir Med. 2010;104: 1809–1816. doi: 10.1016/j.rmed.2010.06.013 20615680 10.1016/j.rmed.2010.06.013

[58] Brown RH, Scichilone N, Mudge B, Diemer FB, Permutt S, Togias A. High-resolution computed tomographic evaluation of airway distensibility and the effects of lung inflation on airway caliber in healthy subjects and individuals with asthma. Am J Respir Crit Care Med. 2001;163: 994–1001. doi: 10.1164/ajrccm.163.4.2007119 11282779 10.1164/ajrccm.163.4.2007119

[59] Macklem PT. Mechanical factors determining maximum bronchoconstriction. Eur Respir J Suppl. 1989;6: 516s–519s. 2803407

[60] Macklem PT, Mead J. Resistance of central and peripheral airways measured by a retrograde catheter. J Appl Physiol. 1967;22: 395–401. 4960137 10.1152/jappl.1967.22.3.395

[61] Venegas JG, Winkler T, Musch G, Vidal Melo MF, Layfield D, Tgavalekos N, et al Self-organized patchiness in asthma as a prelude to catastrophic shifts. Nature. 2005;434: 777–782. doi: 10.1038/nature03490 15772676 10.1038/nature03490

[62] Horsfield K, Cumming G. Morphology of the bronchial tree in man. J Appl Physiol. 1968;24: 373–383. 5640724 10.1152/jappl.1968.24.3.373

[63] Pozin N, Montesantos S, Katz I, Pichelin M, Vignon-Clementel I, Grandmont C. A tree-parenchyma coupled model for lung ventilation simulation. Int J Numer Methods Biomed Eng. 2017; n/a–n/a. doi: 10.1002/cnm.2873 28224760 10.1002/cnm.2873

[64] Montaudon M, Lederlin M, Reich S, Begueret H, Tunon-de-Lara JM, Marthan R, et al Bronchial measurements in patients with asthma: comparison of quantitative thin-section CT findings with those in healthy subjects and correlation with pathologic findings. Radiology. 2009;253: 844–853. doi: 10.1148/radiol.2533090303 19789219 10.1148/radiol.2533090303

[65] Aysola RS, Hoffman EA, Gierada D, Wenzel S, Cook-Granroth J, Tarsi J, et al Airway remodeling measured by multidetector CT is increased in severe asthma and correlates with pathology. Chest. 2008;134: 1183–1191. doi: 10.1378/chest.07-2779 18641116 10.1378/chest.07-2779 PMC2859729

[66] Gupta S, Siddiqui S, Haldar P, Entwisle JJ, Mawby D, Wardlaw AJ, et al Quantitative analysis of high-resolution computed tomography scans in severe asthma subphenotypes. Thorax. 2010;65: 775–781. doi: 10.1136/thx.2010.136374 20805170 10.1136/thx.2010.136374 PMC2975950

[67] Gupta S, Hartley R, Khan UT, Singapuri A, Hargadon B, Monteiro W, et al Quantitative computed tomography-derived clusters: redefining airway remodeling in asthmatic patients. J Allergy Clin Immunol. 2014;133: 729–738.e18. doi: 10.1016/j.jaci.2013.09.039 24238646 10.1016/j.jaci.2013.09.039 PMC3969578

[68] Fain S, Evans M, Granroth J, Newell J, Wenzell S, Gierada D, et al Variability of Quantitative CT Airway Measures of Remodeling D28 IMAGING OBSTRUCTIVE LUNG DISEASES I. American Thoracic Society; 2009 p. A5575 Available: http://www.atsjournals.org/doi/abs/10.1164/ajrccm-conference.2009.179.1_MeetingAbstracts.A5575

[69] Pyrgos G, Scichilone N, Togias A, Brown RH. Bronchodilation response to deep inspirations in asthma is dependent on airway distensibility and air trapping. J Appl Physiol. 2011;110: 472–479. doi: 10.1152/japplphysiol.00603.2010 21071596 10.1152/japplphysiol.00603.2010 PMC3290002

[70] Benfante A, Bellia M, Scichilone N, Cannizzaro F, Midiri M, Brown R, et al Airway distensibility by HRCT in asthmatics and COPD with comparable airway obstruction. COPD. 2013;10: 560–566. doi: 10.3109/15412555.2013.773304 23537326 10.3109/15412555.2013.773304

[71] Tawhai MH, Hunter P, Tschirren J, Reinhardt J, McLennan G, Hoffman EA. CT-based geometry analysis and finite element models of the human and ovine bronchial tree. J Appl Physiol Bethesda Md 1985. 2004;97: 2310–2321. doi: 10.1152/japplphysiol.00520.2004 15322064 10.1152/japplphysiol.00520.2004

[72] Bai TR, Knight DA. Structural changes in the airways in asthma: observations and consequences. Clin Sci Lond Engl 1979. 2005;108: 463–477. doi: 10.1042/CS20040342 15896192 10.1042/CS20040342

