# Supplementary figures and images for: The effect of disease and respiration on airway shape in patients with moderate persistent asthma
Source: PLoS One. 2017 Jul 31;12(7):e0182052. doi: 10.1371/journal.pone.0182052 (PMC5536319; doi:10.1371/journal.pone.0182052)

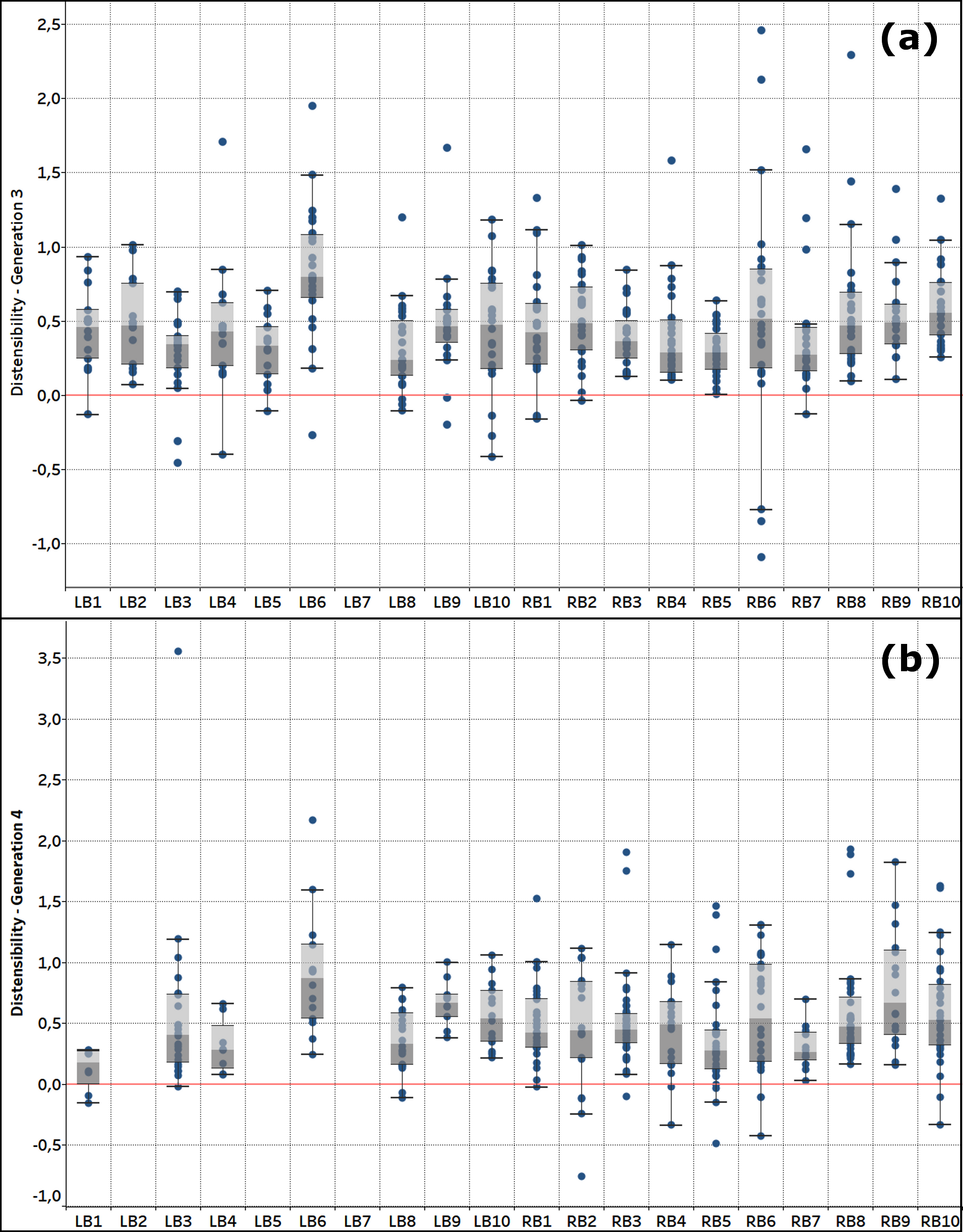

Supplement: S1 Fig — A box-plot of the distensibility for all the available airways of generations 3 and 4. Outliers are observed at above and under 1.5 Inter-Quartile range. (TIF) [file pone.0182052.s001.tif]

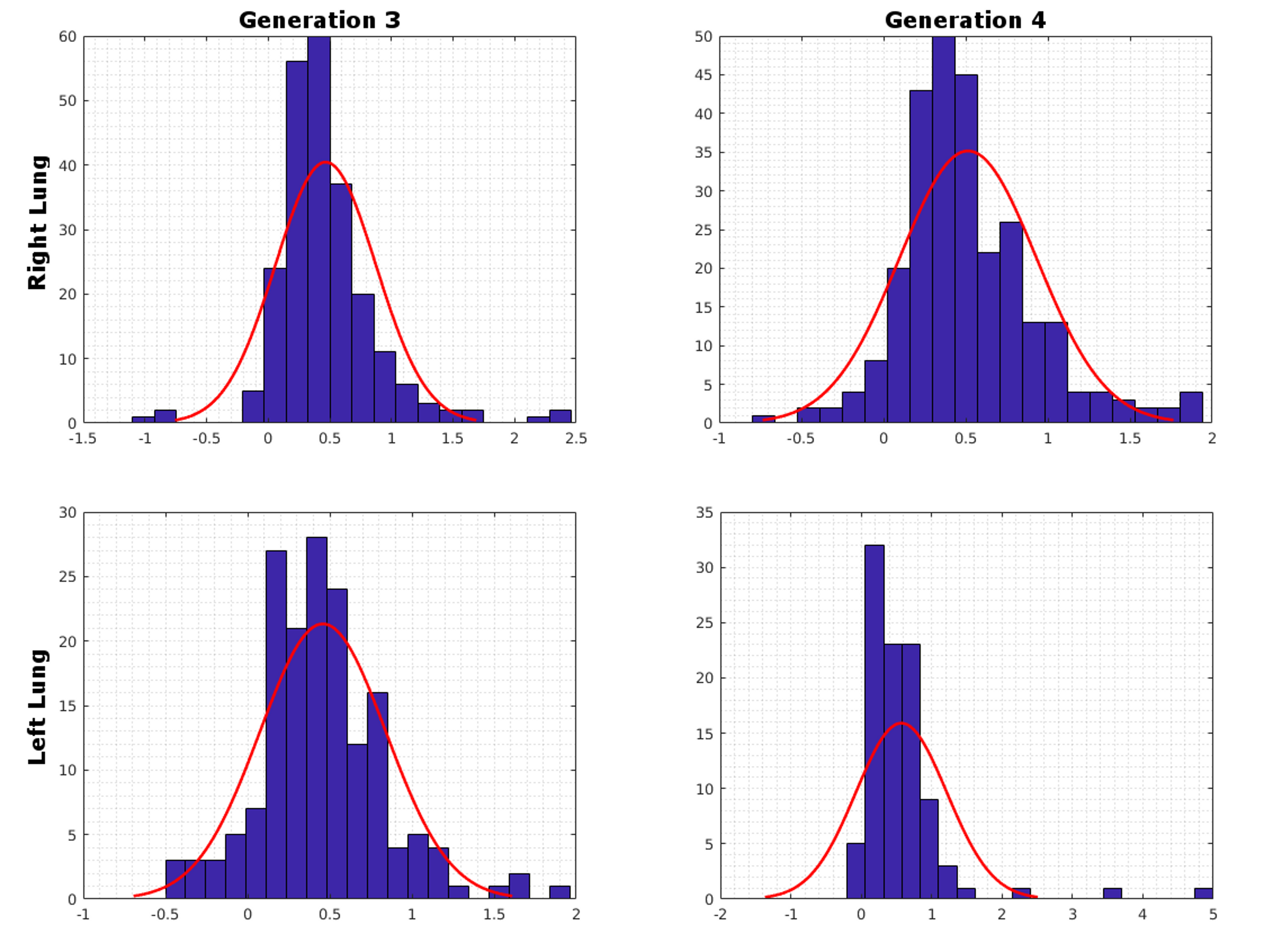

Supplement: S2 Fig — This is a histogram of the distribution of the distensibility data of the right and left lung for the airways of generation 3 and 4. The best-fit normal density function is also shown as a red line. (TIF) [file pone.0182052.s002.tif]

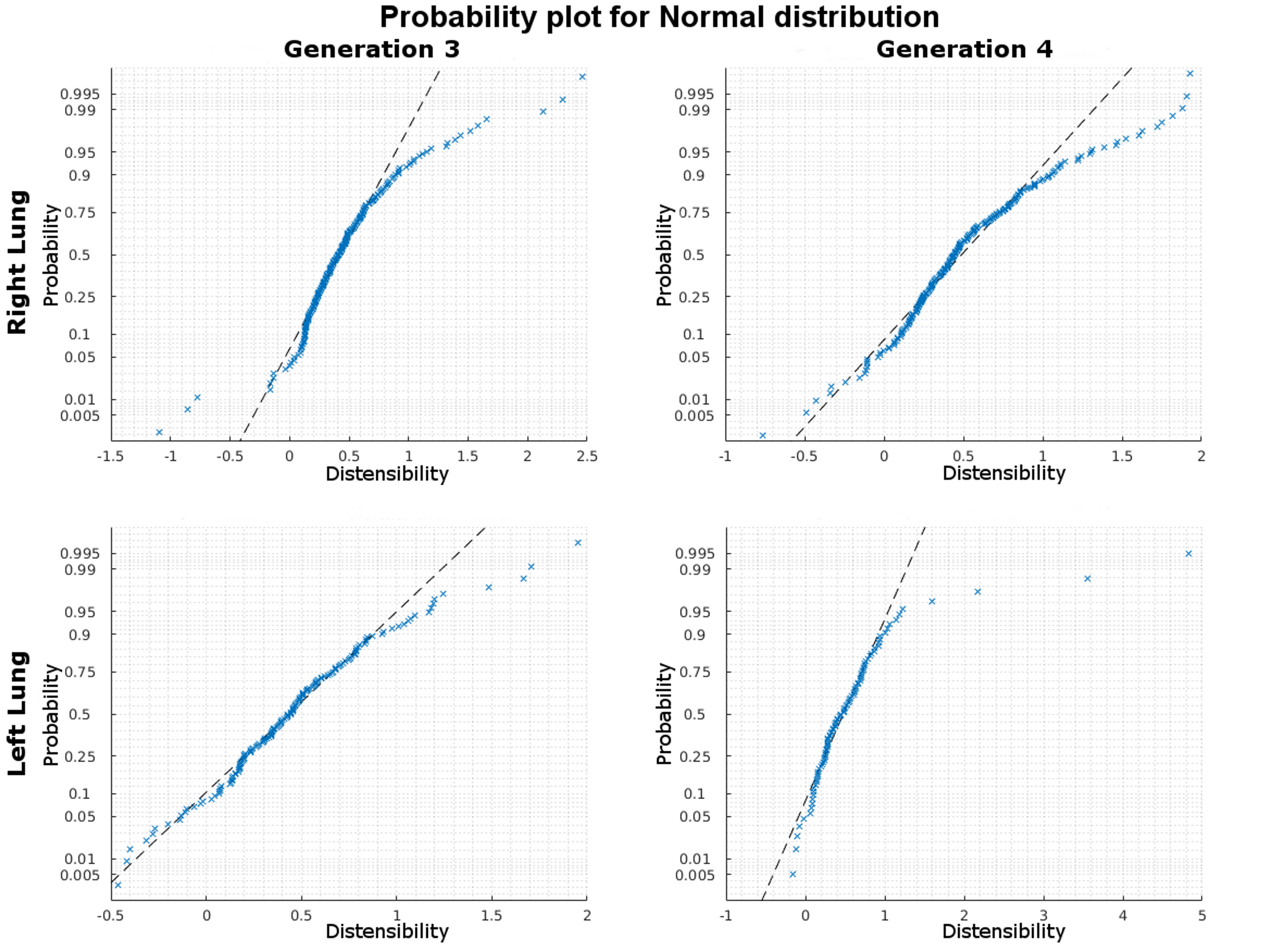

Supplement: S3 Fig — The probability plot comparing the distensibility data to the normal distribution (reference line—-), created for the right and left lung generation 3 and 4 data. It can be observed that the fit of the data to the reference line deteriorates when distensibility values exceed one. (TIF) [file pone.0182052.s003.tif]
