# Supplementary material for: The effect of disease and respiration on airway shape in patients with moderate persistent asthma
Source: PLoS One. 2017 Jul 31;12(7):e0182052. doi: 10.1371/journal.pone.0182052 (PMC5536319; doi:10.1371/journal.pone.0182052)
Supplement: S1 Table — The hydraulic diameter data are normalized to the hydraulic diameter calculated for the trachea and are collected for the same airway tree bronchial regions as the ones considered by Choi et al. [38] (where available). The data are give as average values ± standard deviation values. (DOCX) [file pone.0182052.s004.docx]

| Bronchial Regions | Current study | Choi et al. 2015 | | |
| --- | --- | --- | --- | --- |
|  | Moderate persistent asthmatics | Healthy Subjects | Non-Severe asthmatics | Severe asthmatics |
| R | 0.883±0.063 | 0.828±0.06 | 0.825±0.08 | 0.82±0.09 |
| RU | 0.567±0.073 | 0.542±0.06 | 0.538±0.09 | 0.529±0.11 |
| RMI | 0.646±0.049 | 0.622±0.05 | 0.629±0.06 | 0.645±0.06 |
| RM | 0.397±0.048 | 0.390±0.05 | 0.376±0.06 | 0.358±0.07 |
| RB1+RB2+RB3 | 0.308±0.062 | 0.321±0.03 | 0.300±0.04 | 0.303±0.05 |
| RB4+RB5 | 0.268±0.045 | 0.280±0.04 | 0.261±0.05 | 0.251±0.05 |
| RB6+RB7+RB8+RB9+RB10 | 0.286±0.092 | 0.303±0.04 | 0.281±0.05 | 0.274±0.05 |
| L | 0.691±0.042 | 0.661±0.06 | 0.658±0.06 | 0.684±0.07 |
| LU | 0.589±0.068 | 0.570±0.09 | 0.549±0.06 | 0.557±0.08 |
| LL | 0.582±0.050 | 0.555±0.06 | 0.548±0.07 | 0.55±0.09 |
| LB1+LB2+LB3+LB4+LB5 | 0.262±0.057 | 0.262±0.03 | 0.246±0.03 | 0.248±0.04 |
| LB6+LB8+LB9+LB10 | 0.328±0.05 | 0.338±0.04 | 0.316±0.05 | 0.307±0.05 |
